# The relationship between RASSF1A gene promoter methylation and the susceptibility and prognosis of melanoma: A meta-analysis and bioinformatics

**DOI:** 10.1371/journal.pone.0171676

**Published:** 2017-02-16

**Authors:** Chihao Shao, Wenzhu Dai, Haili Li, Wenru Tang, Shuting Jia, Xiaoming Wu, Ying Luo

**Affiliations:** Laboratory of Molecular Genetics of Aging and Tumor, Medical Faculty; Kunming University of Science and Technology, Kunming, Yunnan, China; Emory University, UNITED STATES

## Abstract

**Background:**

The function of the tumor suppressor gene RASSF1A in cancer cells has been detailed in many studies. However, due to the methylation of its promoter, the expression of RASSF1A is missing in most cancers. In the literature, we found that the conclusion regarding the relationship between RASSF1A gene promoter methylation and the susceptibility and prognosis of melanoma was not unified. This study adopts the use of a meta-analysis and bioinformatics to explore the relationship between RASSF1A gene promoter methylation and the susceptibility and prognosis of melanoma.

**Methods:**

Data on melanoma susceptibility were downloaded from the PubMed, Cochrane Library, Web of Science and Google Scholar databases, which were analyzed via a meta-analysis. The effect sizes were estimated by measuring an odds ratio (OR) with a 95% confidence interval (CI). We also used a chi-squared-based Q test to examine the between-study heterogeneity, and used funnel plots to evaluate publication bias. The data on melanoma prognosis, which were analyzed by bioinformatics methods, were downloaded from The Cancer Genome Atlas (TCGA) project. The effect sizes were estimated by measuring the hazard ratios (HRs) with a 95% confidence interval (CI).

**Results:**

Our meta-analysis included 10 articles. We found that RASSF1A gene promoter methylation was closely related to melanoma susceptibility (OR = 12.67, 95% CI: 6.16 ∼ 26.05, z = 6.90, P<0.0001 according to a fixed effects model and OR = 9.25, 95% CI: 4.37 ∼ 19.54, z = 5.82, P<0.0001 according to a random effects model). The results of the meta-analysis did not reveal any heterogeneity (tau^2^ = 0.00; H = 1 [1; 1.55]; I^2^ = 0% [0%; 58.6%], P = 0.5158) or publication bias (t = 0.87, P = 0.4073 by Egger’s test; Z = 0.45, P = 0.6547 by Begg’s test); therefore, we believe that the results of our meta-analysis were more reliable. To explore the relationship between RASSF1A gene methylation, the prognosis of melanoma and the clinical features of this cancer type, we used the melanoma DNA methylation data and clinical data from TCGA project. We found that RASSF1A gene promoter methylation and melanoma prognosis did not demonstrate any relationship (HR was 0.94 (95% CI = [0.69; 1.27], P = 0.694) with disease-free survival and 0.74 (95% CI = [0.53; 1.05], P = 0.106) for overall survival), and no significant difference was observed between RASSF1A gene promoter methylation and the clinical-pathological features of melanoma.

**Conclusions:**

In conclusion, the meta-analysis of the data in these articles provides strong evidence that the methylation status of the RASSF1A gene promoter was strongly related to melanoma susceptibility. Our bioinformatics analysis revealed no significant difference between RASSF1A gene promoter methylation and the prognosis and clinical-pathological features of melanoma.

## Introduction

Melanoma is a tumor that is produced by melanocytes in the skin. The primary clinical features of skin melanoma are the pigmented lesions that display obvious changes throughout months or years. Despite its low incidence, the malignant degree of this cancer is high, transfer occurs early, and mortality is high; thus, early diagnosis and early treatment are very important. Although malignant melanoma occurs mostly in adults, congenital secondary cancers of giant pigmented nevi are found in children. The outcomes of melanoma development and occurrence are affected by genetic and environmental factors or by a combination of both genes and the environment. Gene changes include DNA promoter methylation, which participates in the early formation of tumors and also plays an important role in the process of tumor development. Because DNA promoter methylation is an important mechanism for tumor suppressor gene inactivation in cancer, the measurement of such methylation could act as a powerful biomarker for the early detection of melanoma. Therefore, we believe that the measurement of DNA promoter methylation may become a powerful tool for the diagnosis of melanoma [1–3].

The Ras association domain family 1 A (RASSF1A) is a 39 kDa protein, while its cDNA is 1859 bp and consists of 6 exons (1α, 2αβ, 3, 4, 5, 6). The Ras-associated region is located at the C terminal end, which may cause weaker interactions with proteins in the Ras superfamily. The C terminal region also contains a Salvador-RASSF1A-Hippo (SARAH) domain. The SARAH domain plays a key role during interactions of RASSF1A with Hippo signaling pathway-related proteins (such as the mammalian sterile 20-like kinase and Salvador). In addition, RASSF1A contains ATM (ataxia telangiectasia mutant) kinase phosphorylation sites, which are located between amino acids 125–138. The terminal region is rich in cysteine resides, which function in RASSF1A-mediated cell apoptosis.

In cells, RASSF1A is an important tumor suppressor gene that can promote apoptosis, control the cell cycle and mitosis, and maintain the stability of microtubules[4–7]. However, in cancer cells, such as breast cancer cells [8], lung cancer cells [9], and liver cancer cells [10], the expression of RASSF1A was demonstrated to be lower, and when RASSF1A was expressed ectopically, the proliferation and migration of cancer cells were suppressed. Previous studies found that the lower expression of RASSF1A in cancer cells was caused by its own promoter methylation. Hence, the relationship between RASSF1A gene promoter methylation and tumor formation/development was revealed in many studies. Although the relationship between RASSF1A gene promoter and breast cancer [11], lung cancer [12], colorectal cancer [13], prostate cancer [14], ovarian cancer [15] and hepatocellular carcinoma [16] had been demonstrated, the relationship between RASSF1A gene promoter and melanoma was not sure. Therefore, we carry out a correlation analysis based on meta-analysis and bioinformatics method to explore the relationship between RASSF1A gene promoter and melanoma.

In our study, we first devised keywords for the retrieval of studies from four literature databases; we then selected the studies that contained cancer samples and normal samples to analyze the relationship between RASSF1A gene promoter methylation and melanoma susceptibility. At the same time, we also tested for heterogeneity and publication bias and in addition performed a sensitivity analysis. We also downloaded the available methylation data and clinical data for melanoma from TCGA project. We used bioinformatics to analyze the relationship between RASSF1A gene promoter methylation and the prognosis of melanoma, and simultaneously, we analyzed the relationship between the frequency of RASSF1A gene promoter methylation and the clinical features of patients with melanoma. Our goal was to explore the relationship between RASSF1A gene promoter methylation and the susceptibility and prognosis of melanoma, as well as whether promoter methylation of the RASSF1A gene might be a biomarker for the clinical detection of melanoma.

## Methods

### Search strategy, selection of studies and data extraction

The design of keywords allowed us to search a range of digital databases, including PubMed, Cochrane Library, Web of Science and Google Scholar, for articles published in English up until May 2016. This study used a subject and text word strategy, as follows: ‘melanoma or melanotic cancer or black cancer’, ‘RASSF1A or Ras association domain family 1 A or RASSF1’, ‘methylation or hyper-methylation or epigenetic’. The included articles satisfy the following criteria: (1) original study and the melanoma patent had to be based on clinical diagnosis; (2) the subjects in every study included melanoma cancer samples and normal controls (blood/tissue of healthy person, or adjacent cancer normal tissue); (3) the RASSF1A methylation frequency had to be include in case and control groups of every study; (4) the papers were written in English. The exclusion criteria were as follows: (1) cell or animal experiments, and letters, review papers, commentaries; (2) no controlled clinical observational studies in the study; (3) the RASSF1A gene promoter region was not contained the CpG island A (737bp, containing 85 CpG dinucleotides) [17–19] (4) not included RASSF1A gene promoter methylation and/or melanoma.

Chihao Shao and Haili Li independently reviewed the selected studies used the above selection standards extracted the data. Decisions were made and any disagreements regarding decisions were resolved by discussion with Wenru Tang and Xiaoming Wu. The following information was extracted from the studies: the first author’s last name of the paper, the year of publication of the paper, age status of the patients, gender status of the patients, TNM.stage of the patients, the original country of the patients, the method used to examine the RASSF1A gene methylation, the number of RASSF1A gene methylations in individual cases and controls, the primers that were used in the article, among other pieces of information.

### Meta-analysis

The data we selected from articles were analyzed and visualized mainly using R (R version 3.2.0) software. The strength of the relationship between RASSF1A gene promoter methylation and the risk of melanoma was measured by the pooled odds ratios (ORs) with a 95% confidence interval (CI). The significance of the pooled OR was determined by the Z-test with a threshold of P<0.05. The Q, H and I^2^ statistic were used to test the heterogeneity. H>1.5, I^2^ values over 50% and Chi-squared test values of P≤ 0.1 showed strong heterogeneity among the studies[20]. To explain the heterogeneity of the subgroup differences, we used Tau-squared (τ^2^) [21]. The data were pooled using a random effects model (H>1.5, I^2^>50%, P≤0.1) or a fixed effects model (H<1.2, I^2^≤50%, P>0.1) according to the heterogeneity statistic I^2^ [21]. When heterogeneity was shown among the included studies, the pooled OR estimates were calculated by a random effects model [21]. Otherwise, a fixed effects model was used [22]. To assess the contributions of single studies to the final results, sensitivity analyses were performed. Generally, Begg’s test and Egger’s test were used to assess funnel plot asymmetry related to reporting publication bias [23, 24]. When Z>1.96, P<0.05 by Begg’s test or P<0.05 according to Egger’s test, we considered the existence of bias, and we used a conventional meta-trim method to re-estimate the effect size.

### The extraction and analysis of TCGA data

Information on DNA methylation in cases of melanoma was collected from TCGA project using methylation 450 K dataset [http://cancergenome.nih.gov/]. We also downloaded clinical information on melanoma from TCGA project. The 450 K dataset contained 485,577 probes, and the methylation status of each probe was defined according to the beta-value as follows: the beta-value = intensity value from the methylated bead types/(intensity values from the methylated bead types + intensity value from the unmethylated bead types + 100). If the beta-value≥0.6, complete methylation was considered. If the beta-value≤0.2, complete unmethylation was considered. If the beta-values were between 0.2 and 0.6, the site was considered to be partially methylated. The CpG site was considered methylated when the probe beta-value was greater than the empirical threshold of 0.3 [25].

Cox proportional hazards regression models were generated for overall survival (OS) and disease-free survival (DFS) calculations, which incorporated multiple variables. Survival curves were constructed using the log-rank method. Chi-square test or Fisher’s exact test was used to examine the relationship between qualitative variables. A P value<0.05 was considered significant.

## Results

### The relationship between RASSF1A gene promoter methylation and melanoma susceptibility

**Fig 1:**
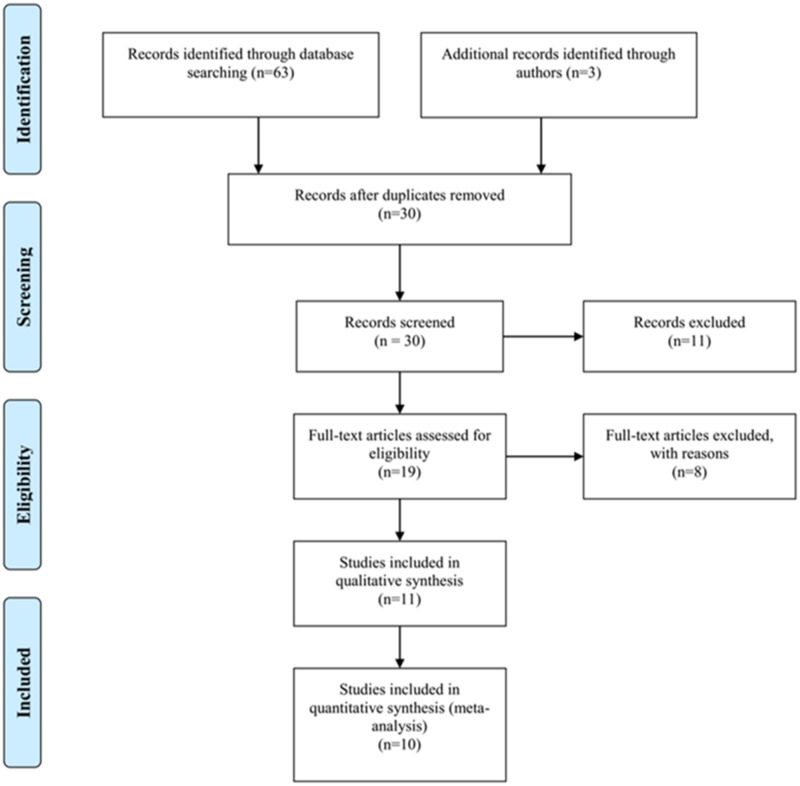
Flow chart of study identification.

The above search strategy resulted in the initial selection of 30 studies. After further screening for inclusion on the basis of their titles, abstracts and full texts, we finally selected 10 studies [26–35] (Fig 1). The characteristics of the 10 articles are shown in Table 1. All of these articles were written in English. In total, 589 melanoma samples and 186 normal control samples were collected. Among the studies that examined the risk of melanoma, two articles that examined blood and 8 articles that examined tissues. In regards to the experimental methods used to explore RASSF1A gene promoter methylation status, 8 of 10 included studies used methylation specific polymerase chain reaction (MSP), while the others used quantitative methylation specific polymerase chain reaction (qMSP, include Methylight, Prosequencing, semi-quantitative fluorescence MSP). The primers used in both methods are listed in Table 2. The promoter region and the CpG sites of the RASSF1A gene were previously described [9, 19, 36].

**Table 1:** Characteristics of eligible studies considered in the report.

Author	Year	Country	Method	Sample	WHO grade	Mean age (year)	Male/ Female	Case		Control	
								M	T	M	T
Calipel et al.	2011	France	MSP	Tissue	NA	NA	NA	9	41	1	9
Tanemura et al.	2009	USA	MSP	Tissue	I-IV	59.4	39/68	35	122	0	35
Liu et al.	2008	USA	QMSP	Tissue	NA	NA	NA	64	100	1	3
Koyanagi et al.	2006	USA	MSP	Blood	IV	45	38/12	16	50	0	40
Marini et al.	2006	Germany	MSP	Tissue	II-IV	60.3	NA	26	41	0	13
Mori et al.	2005	USA	MSP	Blood	I-IV	NA	38/12	13	50	0	50
Reifenberger et al.	2004	Germany	QMSP	Tissue	II-V	67	12/25	9	30	0	3
Hoon et al.	2004	USA	MSP	Tissue	NA	NA	NA	49	86	3	20
Furuta et al.	2004	Japan	MSP	Tissue	III-IV	NA	NA	9	25	0	2
Spugnardi	2003	USA	MSP	Tissue	III-IV	NA	NA	24	44	0	11

MSP, methylation-specific polymerase chain reaction; QMSP, quantitative methylation-specific polymerase chain reaction; T, the total number of patients in each article; M, the number of patients with methylation; NA, not available.

**Table 2:** Three types of primers used in the 10 studies.

Author	Primer (5'-3')	Primer Types	Genomic Coordinate
Koyanagi et al.	F:GTGTTAACGCGTTGCGTATC	Primer located II	chr3:50353454–50353548
	R:AACCCCGCGAACTAAAAACGA		
Calipel et al.	F:GGGTTTTGCGAGAGCGCG	Primer located I	chr3:50353037–50353206
	R:GCTAACAAACGCGAACCG		
Tanemura et al.	F:GTGTTAACGCGTTGCGTATC	Primer located II	chr3:50353454–50353548
	R:AACCCCGCGAACTAAAAACGA		
Liu et al.	F:GTGTTAACGCGTTGCGTATC	Primer located II	chr3:50353454–50353548
	R:AACCCCGCGAACTAAAAACGA		
Marini et al.	F:ATTGAGTTGCGGGAGTTGGT	Primer located III	chr3:50353190–50353281
	R:ACACGCTCCAACCGAATACG		
Mori et al.	F:GGGTTTTGCGAGAGCGCG	Primer located I	chr3:50353037–50353206
	R:GCTAACAAACGCGAACCG		
Reifenberger et al.	F:CCTCTGTGGCGACTTCATCTG	Primer located III	chr3: 50352808–50353544
	R:CAACAGTCCAGGCAGACGAG		
Hoon et al.	F:GTGTTAACGCGTTGCGTATC	Primer located II	chr3:50353454–50353548
	R:AACCCCGCGAACTAAAAACGA		
Furuta et al.	F:GTCGTCGTTGTGGTCGTTC	Primer located II	chr3:50353454–50353548
	R:AACCCGAAAACGAAACTAAACG		
Spugnardi et al.	F:GGGTTTTGCGAGAGCGCG	Primer located I	chr3:50353037–50353206
	R:GCTAACAAACGCGAACCG		

F, Forward primers; R, Reverse primers; NA, not available.

**Fig 2:**
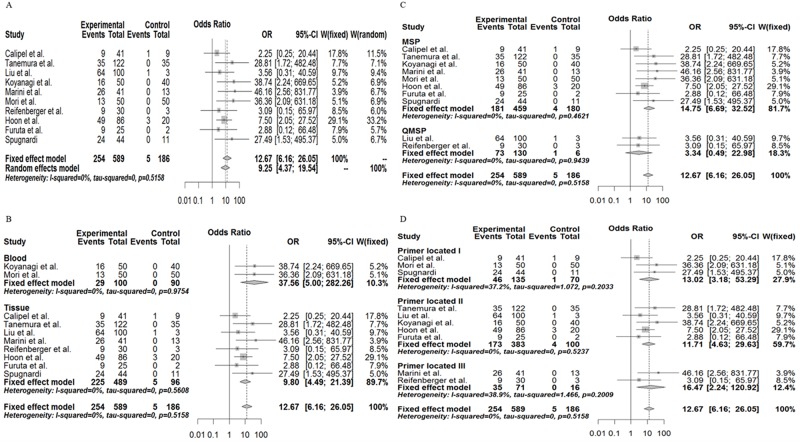
Combined estimates of the association between RASSF1A gene promoter methylation and melanoma susceptibility with a forest plot. (A), Meta-analysis of **t**he association between RASSF1A gene promoter methylation and melanoma susceptibility by a random effects model and a fixed effects model. (B), Subgroup meta-analysis based on race by a random effects model and a fixed effects model. (C), Subgroup meta-analysis based on different methylation detection methods by a random effects model and a fixed effects model. (D), Subgroup meta-analysis based on different primer types by a random effects model and a fixed effects model.

According to the meta-analysis, we found that the frequency of RASSF1A gene promoter methylation in melanoma was: OR = 12.67, 95% CI: 6.16∼26.05, z = 6.90, P<0.0001 by a fixed effects model and OR = 9.25, 95% CI: 4.37∼19.54, z = 5.82, P<0.0001 by a random effects model without evidence of heterogeneity (tau^2^ = 0.00; H = 1 [1; 1.55]; I^2^ = 0% [0%; 58.6%], P = 0.5158). This shows a statistically significant increase in the likelihood of RASSF1A gene promoter methylation in melanoma compared with the controls (Fig 2A). Although the tau^2^ was very small, we also observed a difference in the various subgroups. As we have speculated, significant differences were also found among the tissue samples (OR = 9.80, 95% CI: 4.49∼21.39), and blood samples (OR = 37.56, 95% CI: 5.00∼288.26, P<0.0001) (by a fixed effects model) (Fig 2B). As we have inferred, the different methods used to assess methylation (both MSP and QMSP) also revealed a significant association between RASSF1A gene methylation and melanoma (OR = 14.75, 3.34 by a fixed effects model, respectively) (Fig 2C), as association was also shown with the use of different primers (Fig 2D). Hence, the methylation status of the RASSF1A gene in either tissue or serum samples may be used as a potential biomarker for melanoma diagnosis.

**Fig 3:**
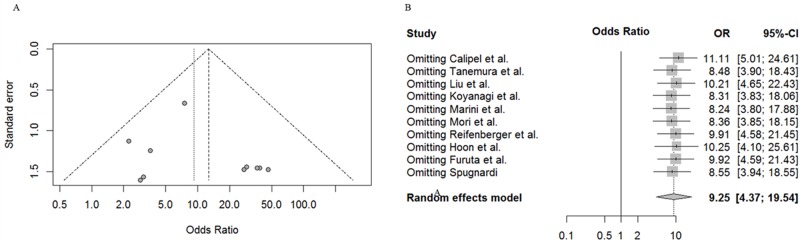
Funnel plot for publication bias test and sensitivity analysis of the summary odds ratio coefficients on the relationship between RASSF1A gene promoter methylation and melanoma.

To assess the publication bias of the 10 articles, we used Begg’s test and Egger’s test. As we have inferred, the assessment of Egger’s test and Begg’s test did not reveal any publication bias in the ensemble analysis (t = 0.87, P = 0.4073; Z = 0.44, P = 0.6547) (Fig 3A). In regards to the sensitivity analysis, the results ranged from 8.24 (95% CI, 3.80∼17.88) to 11.11 (95% CI, 5.01∼24.61) upon the omission of a single study with the random effects model (Fig 3B). Hence, as indicated by the sensitivity analysis, Begg’s test and Egger’s test, the credibility and accuracy of our meta-analysis result was very high.

### The relationship between RASSF1A gene promoter methylation and prognosis of patients with melanoma

According to previous studies [9, 19, 36] and assembly-Mar.2006 (NCBI 36/hg18), we analyzed 11 different probes located in the RASSF1A gene promoter region (cg24859722, cg13872831, cg00777121, cg04743654, cg12966367, cg08047457, cg25747192, cg21554552, cg27569446, cg25486143, cg06172942), which contain the transcription start site (chr3:50353240) of the RASSF1A gene and the CpG island A (chr3: 50352808–50353544) of RASSF1 gene. We collected data from 355 patients with skin cutaneous melanoma from TCGA project for a correlation analysis. The ages of the 365 patients with skin cutaneous melanoma ranged from 15 to 90, and the American Joint Committee on Cancer (AJCC) pathologic tumor stage ranged from 0-IV. Moreover, 221 patients were male, and 134 were female (S1 Table). In regards to the relationship between RASSF1A gene promoter methylation and the disease-free survival (DFS) of patients with skin cutaneous melanoma, we chose 314 patients for analysis. In terms of the relationship between RASSF1A gene promoter methylation and overall survival (OS) of patients with skin cutaneous melanoma, we selected 350 patients for analysis. All the patients with skin cutaneous melanoma who were chosen by us received surgical treatment. Of the 314 patients with skin cutaneous melanoma selected for the DFS analysis, 31.52% had RASSF1A gene promoter methylation. Among the 350 patients with skin cutaneous melanoma selected for the OS analysis, 32.00% of patients had RASSF1A gene promoter methylation.

**Fig 4:**
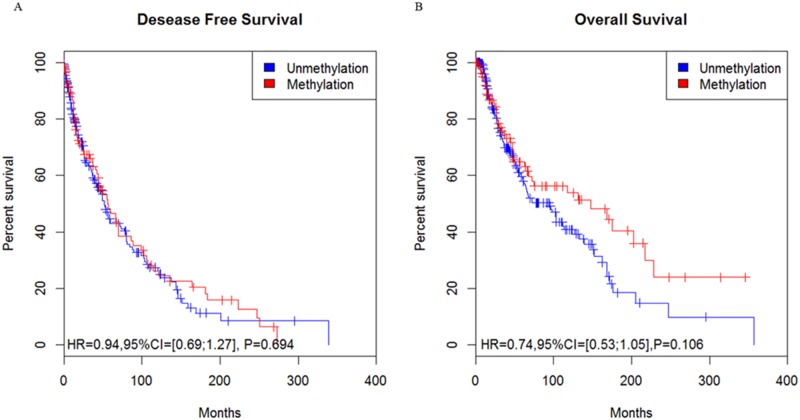
The relationship of RASSF1A gene promoter methylation and the survival curve of patients with skin cutaneous melanoma from TCGA data. (A, B), Association of patient survival and RASSF1A gene methylation status based on the Kaplan-Meier method.

After we used the Kaplan-Meier method to analyze the relationship between RASSF1A gene promoter methylation and the prognosis of patients with skin cutaneous melanoma, we found that the HR was 0.94 (95% CI = [0.69; 1.27], P = 0.694) for the DFS (Fig 4A) and 0.74 (95% CI = [0.53; 1.05], P = 0.106) for the OS (Fig 4B), which suggests that no significant difference existed between RASSF1A gene promoter methylation and the prognosis of patients skin cutaneous melanoma.

We also used the data from TCGA project to explore the relationship between RASSF1A gene promoter methylation and the clinical features of patients with skin cutaneous melanoma. As we speculated, no statistically significant difference was found between RASSF1A gene promoter methylation and patient age (the P value was 0.514 for age>60 vs age≤60), AJCC pathologic tumor stage (the P value was 0.939 for stage 0-II vs stage III-IV), or gender (the P value was 0.355 for males vs females).

## Discussion

Melanoma generally refers to malignant melanoma, which is a type of skin malignancy. Melanoma, squamous cell carcinoma and basal cell carcinoma are the three most common malignant tumors of the skin. Melanoma forms when melanocytic nevi or melanin spots become malignant. In recent years, melanoma has become the fastest growing malignant tumor among all malignant tumors, and its annual growth rate is 3% to 5%. More seriously, when malignant melanoma is in its rapid growth period, the prognosis of patients is poor and the mortality rate is higher.

The formation and development of tumors are the result of the combination of external and internal factors. External factors include chemical agents (e.g., alkylating agents, polycyclic aromatic hydrocarbons), physical factors (e.g., ionizing radiation), and biological factors (e.g., viruses). Internal factors include genetic, immune and endocrine factors. Genetic factors such as mutations and epigenetic changes (e.g., DNA promoter methylation) play an important role in the activation of gene transcription. RASSF1A, which is an important tumor suppressor gene in cells, functions in apoptosis, cell cycle control, and microtubule stability. Dammann et al.[9] discovered and investigated the cellular function of RASSF1A, and the present study confirmed that a lower expression of RASSF1A was mainly caused by its abnormal promoter methylation. Our study used a meta-analysis and bioinformatics first to explore the relationship between RASSF1A gene promoter methylation and melanoma.

Our meta-analysis included 10 articles. We found that RASSF1A gene promoter methylation was closely related to melanoma susceptibility. According to the results of the meta-analysis, heterogeneity and publication bias were not present, and therefore, we believe that our meta-analysis results were reliable. However, there were two limitations in this meta-analysis as we regarded. Firstly, the number of normal samples were too small in some articles [28, 34], thereby limiting confidence in drawing conclusions. Secondly, the meta-analysis was short of sufficient statistical power to assess the correlations between RASSF1A gene promoter methylation and difference status (such as age, gender and pathologic tumor stage) of melanoma. Considered the above limitations, we used the melanoma DNA methylation data and clinical data from TCGA project in order to further explore the relationship between RASSF1A gene methylation and melanoma. However, considered there was no normal control in the melanoma of TCGA project, we only focused on the prognosis of melanoma and the clinical features of patients with melanoma. Ultimately, we found no relationship between RASSF1A gene promoter methylation and melanoma prognosis, and no significant difference between RASSF1A gene promoter and the clinical-pathological features of melanoma.

Even though our results are reliable, when a separate analysis of the relationship between a single gene at one molecular level and cancer is conducted, some shortcomings would inevitably result. Therefore, the next step should be a comprehensive analysis of the relationship between multiple genes at all molecular levels and cancer, so that better guidance may be provided for the initial clinical cancer diagnosis and treatment.

In conclusion, this meta-analysis of article data provides strong evidence that the methylation status of the RASSF1A gene promoter was strongly related to melanoma susceptibility. Additionally, we firstly used the bioinformatics analysis focused on the relationship between RASSF1A gene promoter and melanoma. However, we found no significant difference between RASSF1A gene promoter methylation and the prognosis or between RASSF1A promoter methylation and the clinical-pathological features of melanoma.

## Supporting information

S1 PRISMA Checklist (DOCX)

S1 Table Characteristics of the data obtained from the TCGA project. (XLSX)
