# Supplementary material for: The relationship between RASSF1A gene promoter methylation and the susceptibility and prognosis of melanoma: A meta-analysis and bioinformatics
Source: PLoS One. 2017 Feb 16;12(2):e0171676. doi: 10.1371/journal.pone.0171676 (PMC5312935; doi:10.1371/journal.pone.0171676)
Supplement: S1 PRISMA Checklist — (DOCX) [file pone.0171676.s001.docx]

|  | Item | Section name and paragraph number within manuscript |
| --- | --- | --- |
|  | **Introduction** |  |
| 1 | Include sufficient scientific background (including relevant references to previous work) to understand the motivation and context for the study. | 4-5 |
| 2 | Provide an explicit statement of questions being addressed with reference to participants, interventions, comparisons, outcomes, and study design. | 5 |
|  | **Methods** |  |
| 3 | Specify study characteristics and report characteristics (e.g., years considered, language, publication status) used as criteria for eligibility, giving rationale. | 5 |
| 4 | Describe all information sources (e.g., databases with dates of coverage, contact with study authors to identify additional studies) in the search and date last searched. | 5/6-7 |
| 5 | Present full electronic search strategy for at least one database, including any limits used, such that it could be repeated. | 5 |
| 6 | State the process for selecting studies (i.e., screening, eligibility, included in systematic review, and, if applicable, included in the meta‐analysis). | 5-6 |
| 7 | Describe method of data extraction from reports (e.g., piloted forms, independently, in duplicate) and any processes for obtaining and confirming data from investigators. | 5-6 |
| 8 | List and define all variables for which data were sought and any assumptions and simplifications made. | 5 |
| 9 | Describe methods used for assessing risk of bias of individual studies. | 6 |
| 10 | State the principal summary measures (e.g., risk ratio, difference in means). | 6 |
| 11 | Describe the methods of handling data and combining results of studies, if done, including measures of consistency (e.g., I2) for each meta‐analysis. | 6 |
| 12 | Specify any assessment of risk of bias that may affect the cumulative evidence (e.g., publication bias, selective reporting within studies). | 6 |
| 13 | Describe methods of additional analyses (e.g., sensitivity or subgroup analyses, meta-regression), if done, indicating which were pre‐specified. | 6 |
|  | **Results** |  |
| 14 | Include flow diagram for the studies included in the meta-analysis as the first figure for the manuscript | 7-8/9 |
| 15 | For all outcomes considered (benefits or harms), present, for each study: (a) simple summary data for each intervention group (b) effect estimates and confidence intervals, ideally with a forest plot (c) the result of TCGA dataset | 7-9 |
| 16 | Report the effect size estimates and p values for each analysis. | 7-9 |
|  | **Discussion** |  |
| 17 | Summarize the main findings including the strength of evidence for each main outcome. | 9-10 |
| 18 | Give a cautious overall interpretation of results considering objectives, multiplicity of analyses, and other relevant evidence. Meanwhile, Discuss the significance (external validity) of the study results. | 10 |
| 19 | Provide a general interpretation of the results in the context of other evidence, and implications for future research. | 10 |
